# Xanthogranulomatous pancreatitis: A rare entity in the spectrum of pancreatic lesions, a case report

**DOI:** 10.1016/j.ijscr.2024.109810

**Published:** 2024-05-29

**Authors:** Melisa Erina Abdala Bolcatto, Facundo Ignacio Mandojana, Andres Vladimir Verberck Simondi, German Rodrigo Viscido

**Affiliations:** Clinica Universitaria Reina Fabiola, Universidad Catolica de Cordoba, Argentina

**Keywords:** Xanthogranulomatous pancreatitis, Foamy histiocytes, Cephalic duodenopancreatectomy

## Abstract

**Introduction:**

Xanthogranulomatous pancreatitis (XGP) is a rare, benign, and idiopathic disease that often presents with non-specific symptoms and can mimic or coexist with other pancreatic diseases. Despite its infrequency, XGP is frequently misdiagnosed as a pancreatic neoplasm, with only 15 reported cases in the literature. The pathogenesis of XGP remains unclear.

**Case report:**

We present the case of a 34-year-old woman with no pathological history who experienced continuous abdominal pain and oral intolerance, without signs of cholestasis. An abdominal CT scan initially suggested a cystic neoplasm of the pancreas, leading to a laparotomic cephalic duodenopancreatectomy. The anatomopathological study and immunohistochemistry revealed XGP in association with a mucinous cystic neoplasm with mild to moderate atypia. The patient remained hospitalized for six days post-surgery without any complications.

**Discussion:**

XGP may be induced by the inflammatory reaction secondary to the obstruction of the pancreatic duct by mucin. The etiology is unknown, but it is attributed to a combination of obstruction, hemorrhage, or ductal infection. Abdominal pain is the most common symptom. Differentiating XGP from malignant processes of the pancreatic gland is challenging. Surgical treatment typically involves the Whipple procedure; however, echoendoscopy with biopsy is now available for a more accurate and early differential diagnosis.

**Conclusion:**

XGP is a rare and challenging differential diagnosis for pancreatic neoplasms. Due to its potential to mimic malignant lesions, a high index of suspicion is necessary. Echoendoscopy with fine-needle aspiration biopsy should be considered a routine diagnostic tool before major surgery, such as the Whipple procedure.

## Highlights

• Xanthogranulomatous pancreatitis (XGP) is a rare form of chronic inflammation of the pancreatic duct resulting from its obstruction by mucin.
• Abdominal pain is the most common symptom. Other less frequent symptoms include weight loss, acute pancreatitis, or jaundice.
• The diagnosis is usually challenging because imaging studies can confuse this benign entity with a malignant process due to the absence of pathognomonic characteristics
• XGP is documented in deferred anatomopathological and immunohistochemistry studies.
• Given its potential to mimic malignant lesions, a high index of suspicion is necessary.
• Surgical treatment typically involves the Whipple procedure.

## 1 Introduction

Xanthogranulomatous pancreatitis (XGP) is a rare form of chronic pancreatitis characterized by the deposition of numerous foamy histiocytes in the pancreatic parenchyma, along with other inflammatory cells, cholesterol, and fibroblastic proliferation [1]. The etiology of XGP is currently unknown, but it is suspected to involve a combination of ductal obstruction, infection, and repeated intraductal bleeding [2]. To date, in our knowledge there are 15 cases that have been reported in the literature up to 2022 (Table 1). In all of these cases, lesions that mimicked malignancy in imaging studies such as CT scans or magnetic resonance imaging (MRI) were discovered, leading to subsequent surgical treatment [1,[3], [4], [5]]. Differential diagnoses include pancreatic pseudocyst, cystic mucinous neoplasm, and solid pseudopapillary neoplasm [1,2].

**Table 1:** Cases of XGP reported in literature up to 2022.

Authors	Year	Country	Sex	Age (years)	Time of evolution	Symptoms	Size	Pancreatic location	Diagnosis	Treatment	Complications	Recurrence	Survival
Srigley et al.	1993	Canada	M	46	6 months	Jaundice, weight loss, malaise	6 cm	Head	XGP	Pancreaticoduodenectomy	None	None	6 months
Giménez et al.	1998	Spain	M	61	Not specified	Abdominal pain, weight loss, nausea	8 cm	Head	XGP	Distal pancreatectomy, splenectomy	None	None	18 months
Pal et al.	2000	USA	F	36	2 years	Abdominal pain, jaundice	3 cm	Body	XGP	Whipple procedure	None	None	24 months
Singla et al.	2003	India	M	22	2 months	Abdominal pain, jaundice	2.5 cm	Head	XGP	Whipple procedure	None	None	12 months
Singh et al.	2004	India	M	60	1 year	Jaundice, weight loss, fatigue	4 cm	Head	XGP	Whipple procedure	None	None	24 months
Ogawa et al.	2007	Japan	M	69	1 month	Jaundice, abdominal pain, fever	4.5 cm	Body and tail	XGP	Distal pancreatectomy, splenectomy	Portal vein thrombosis	None	7 months
Loh et al.	2008	Australia	M	57	1 year	Jaundice, weight loss	3 cm	Head	XGP	Whipple procedure	None	None	9 months
Ng et al.	2009	Singapore	F	42	3 months	Abdominal pain, weight loss, nausea	3.5 cm	Head	XGP	Whipple procedure	None	None	12 months
Hamaloglu et al.	2009	Turkey	M	38	Not specified	Abdominal pain, weight loss	3 cm	Head	XGP	Whipple procedure	None	None	8 months
Sato et al.	2011	Japan	M	78	Not specified	Abdominal pain, jaundice, weight loss	4 cm	Head	XGP	Whipple procedure	None	None	9 months
Kim et al.	2012	Korea	F	64	Not specified	Abdominal pain, weight loss	1.8 cm	Head	XGP	Whipple procedure	None	None	36 months
Lin et al.	2013	Taiwan	F	75	1 year	Abdominal pain, weight loss, nausea	2 cm	Head	XGP	Whipple procedure	None	None	6 months
Goenka et al.	2015	India	M	56	2 years	Abdominal pain, weight loss, nausea	5 cm	Head	XGP	Whipple procedure	None	None	9 months
Uemura et al.	2017	Japan	F	57	Not specified	Abdominal pain, jaundice	3 cm	Body and tail	XGP	Distal pancreatectomy, splenectomy	None	None	18 months
Talukdar et al.	2019	India	F	34	Not specified	Abdominal pain, weight loss, jaundice	3 cm	Head	XGP	Whipple procedure	None	None	12 months

The objective of this case is to highlight the simultaneous presence of two pathologies, the xanthogranulomatous pancreatitis and the cystic mutinous neoplasm, which is rare and its challenges in diagnosis.

## 2 Methods

This case report has been reported in line with the SCARE criteria [6].

## 3 Case presentation

We present the case of a 34-year-old female patient with no personal pathological or surgical history, BMI of 23 kg/m^2^ who presented to the emergency service of our private university clinic with a three-day history of abdominal pain located on the left flank, of moderate intensity, associated with oral intolerance, and with a history of similar episodes leading to significant weight loss of 7 kg in six months. In the admission laboratory, lipase was 256 U/l (NV 12–60 U/l), total bilirubin 0.52 mg/l (NV < 1 mg/l), aspartate aminotransferase (AST) 33 U/l (NV < 40 U/l), alanine aminotransferase (ALT) 16 U/l (NV < 41 U/l), C-Reactive protein 2 mg/dl (NV < 5 mg/dl), and leukocytes 10 × 10·3/μl (NV 3.8–10 × 10^3^/μl).

**Fig. 1:**
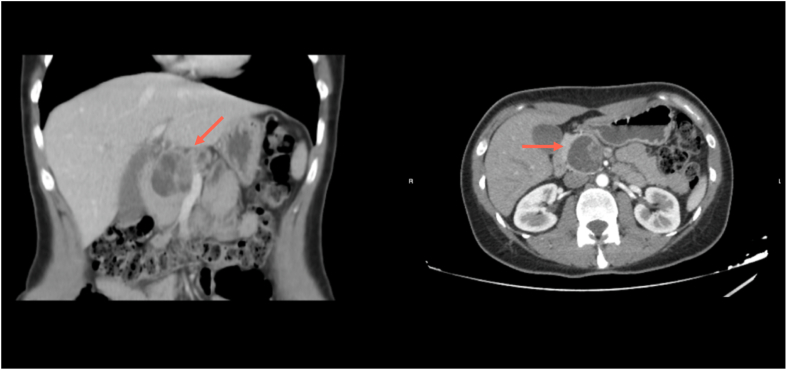
CT scan in which we observe the lesion of the pancreatic head.

An abdominal ultrasound revealed a round image at the pancreatic head, with hypoechoic areas, measuring 4.6 × 3.9 cm. The ultrasound suggested a cystic lesion. This finding was further investigated with an abdominal CT scan with intravenous contrast, revealing a rounded, macrocystic lesion with defined edges, multitabulated, approximately 38 × 38 × 45.8 mm in diameter, located at the head of the pancreas. This lesion generated a slight mass effect on the pancreas and adjacent structures, without evidence of invasion of neighboring vascular structures. Dilation of the Wirsung duct was observed in its corporal and caudal portions with a maximum diameter of 8.3 mm (Fig. 1). Then it was decided to add additional blood work with tumor markers, CA 19-9 of 18 U/ml (NV < 37 U/ml) and carcinoembryonic antigen (CEA) of 2.3 ng/ml (NV < 3.8 ng/ml).

**Fig. 2:**
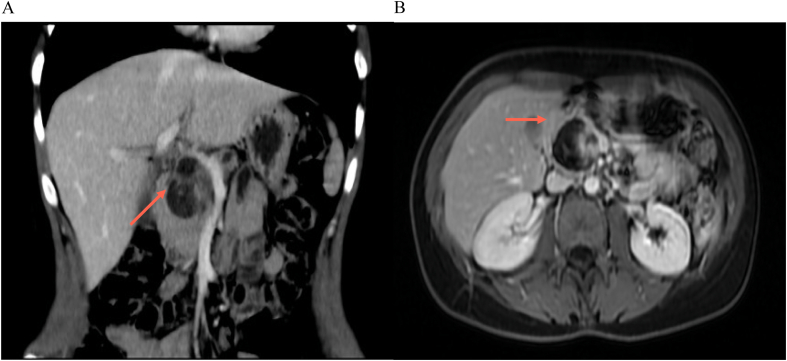
A: MRI in which we observe the cystic multitabulated lesion and the Wirsung duct dilation. B: MRI in which we observe the cystic multitabulated lesion and the Wirsung duct dilation.

**Fig. 3:**
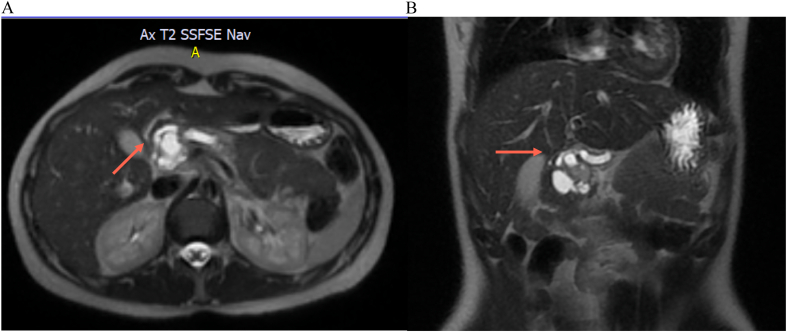
A: MRI in which we observe the Wirsung dilatation and the atrophy of the pancreatic body and tail. B: MRI in which we observe the Wirsung dilatation and the atrophy of the pancreatic body and tail.

Additional imaging studies were conducted with magnetic resonance imaging (MRI) of the abdomen, revealing a cystic lesion with defined edges, heterogeneous content, multitabulated, with thick septa inside, and a 17-mm solid-appearing parietal image with reinforcement in the head of the pancreas. Post-contrast enhancement and restriction in diffusion sequences were observed in this 40 × 38 × 44 mm lesion, along with dilation of the Wirsung duct and atrophy of the body and tail of the pancreas (Figs. 2A B, 3A B).

**Fig. 4:**
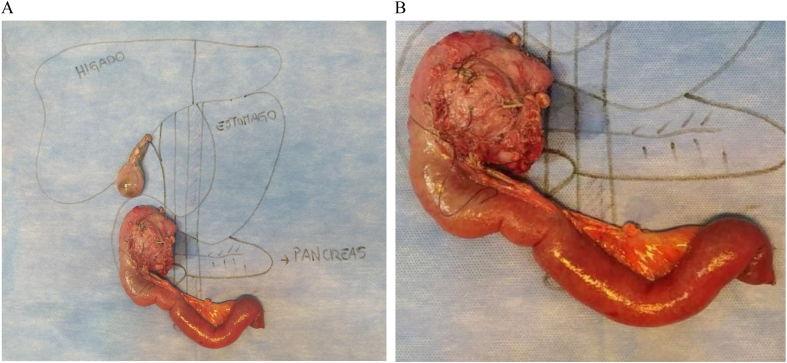
A: Surgical piece of the Whipple procedure. B: Surgical piece of the Whipple procedure.

Due to the high suspicion of malignancy and the persistence of pain and vomiting, a cephalic pancreaticoduodenectomy (Whipple procedure) was performed by laparotomy, revealing a tumor occupying the pancreatic head without extrapancreatic disease (Fig. 4A B). A sample from the distal pancreatic margin was taken for a frozen study, which reported negative results for tumor cells. The procedure lasted 4.5 h without immediate complications. Drains were removed on the 5th post-surgical day after confirming normal amylase and bilirubin values, and the patient was subsequently discharged the next day.

**Fig. 5:**
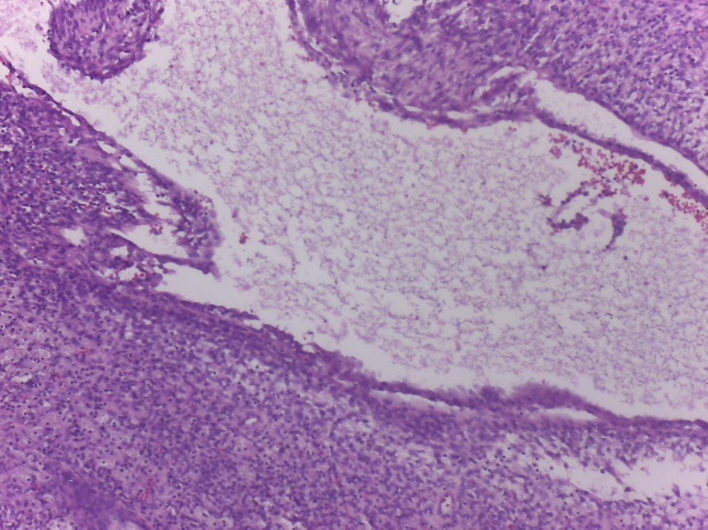
histology of the pancreatic cyst surrounded by macrophage cells.

**Fig. 6:**
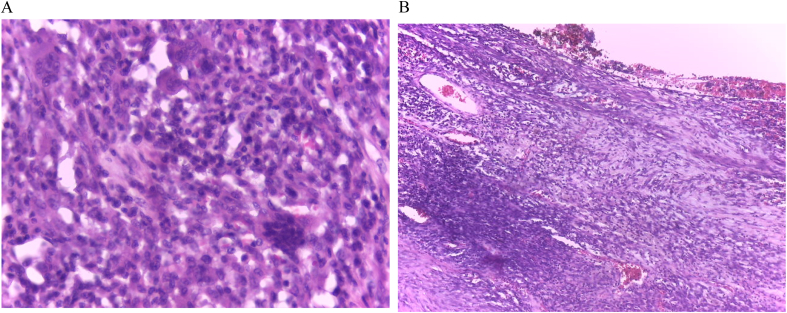
A: Histology of pancreatic parenchyma of multinucleated giant cells. B: Histology of pancreatic parenchyma of multinucleated giant cells and foamy histiocytes.

**Fig. 7:**
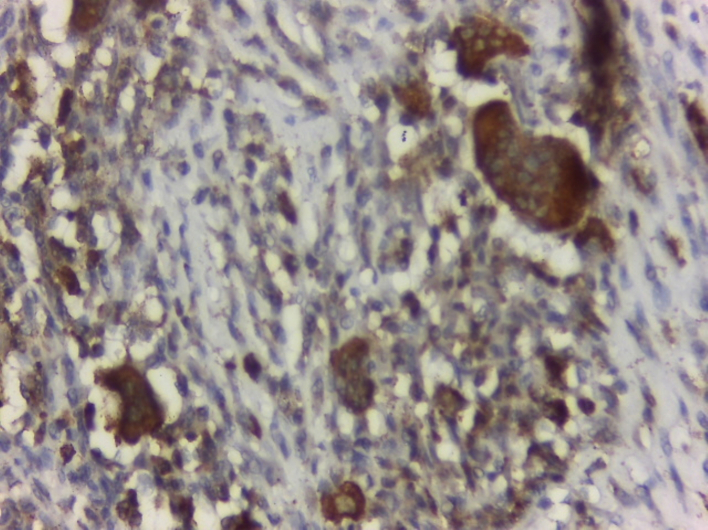
CD68+ immunostaining.

The final pathology report described a neoplastic proliferation composed of spindle cells with mild to moderate atypia, a variably dense stroma, frequent giant cells, and foamy histiocytes. The lesion was interspersed with the pancreatic parenchyma and dilated ducts (Fig. 5, Fig. 6A B). An immunostaining study confirmed xanthogranulomatous pancreatitis with a CD68+ marker in relation to a cystic mucinous neoplasm (Fig. 7). The patient remained asymptomatic and in excellent general condition, with no evidence of recurrence, at the 24-month follow-up as of date of follow-up.

## 4 Discussion

Xanthogranulomatous pancreatitis is a rare form of chronic inflammation of the pancreatic duct resulting from its obstruction by mucin. Although its etiology remains unknown, a combination of ductal obstruction, infection, and repeated bleeding is suspected to contribute to its development [2]. It is characterized by the deposition of foamy histiocytes in the pancreatic parenchyma, along with the proliferation of fibroblasts and other inflammatory cells, and may also involve necrosis and hemorrhage [2]. XGP typically affects men more frequently and presents around the age of 60; however, in our case, the patient was a young adult woman [1]. Abdominal pain is the most common symptom, as reported in this case and in other studies [[1], [2], [3]]. Other less frequent symptoms include weight loss, acute pancreatitis, or jaundice [7], which were not observed in our patient.

The diagnosis is usually challenging because imaging studies can confuse this benign entity with a malignant process due to the absence of pathognomonic characteristics [3]. The differential diagnosis should be made with pancreatic pseudocyst, mucinous cystic neoplasm, neuroendocrine tumor, intraductal papillary mucinous neoplasm, and solid pseudopapillary neoplasm [1,2]. Unlike XPG, mucinous cystic neoplasm occurs more frequently in females [8] and is located in the pancreatic body and tail [9]. The initial treatment in all reported cases was surgical after an imaging diagnosis [1,[3], [4], [5]], and it should be reserved for cases of adenocarcinoma or lesions with a high suspicion of malignancy, as is the case with this entity. Due to this challenging diagnosis, XGP is documented in deferred anatomopathological and immunohistochemistry studies.

Echoendoscopy (EcoE) has emerged as a promising method for the diagnosis of XGP. This minimally invasive procedure combines endoscopy and ultrasound to obtain high-resolution images of the pancreas and surrounding structures. EcoE allows for the visualization of small lesions and fine-needle aspiration biopsy (FNAB) of suspicious areas, which can then be sent for pathological analysis. Studies have reported a sensitivity of 92–100 % and specificity of 89–100 % for EcoE with FNAB in the diagnosis of Xanthogranulomatous pancreatitis, making it a valuable tool for clinicians [1,8]. EcoE with FNAB has also demonstrated high diagnostic accuracy due to the location of the tumor in relation to the duodenal papilla, allowing for an early diagnosis of this entity [8]. However, it is important to note that EcoE with FNAB is a highly specialized and expensive procedure that may not be available in all healthcare settings [10], in this case in particular, we hadn't have access to EcoE, however this study would not have change the outcome, because our patient remained with pain and oral intolerance.

Kwon et al., described the findings on imaging, showing that the most frequent main composition of the XGP-associated mass was cystic in 60 % of their cases with an irregular thick wall, located in the pancreatic tail. The XGP appeared heterogeneous on the portal-phase of CT and MRI. On contrast-enhanced dual - phase CT, the solid component of all lesions showed hypoenhancement on the arterial phase in comparison with the normal pancreas. This was also identified on dynamic contrast - enhanced MRI where lesions were mostly hypointense or isointense, and hyperintense on the delayed phase [12].

On the other hand, Whipple surgery is considered the primary treatment option for pancreatic mucinous adenomas, particularly when they are situated in the pancreatic head, have a considerable size, and display dilation of the pancreatic duct. This is due to the high risk of malignancy, which ranges from 10 to 39 %. In our patient's case, aside from the 2.5 cm lesion in the pancreatic head, pancreatic duct dilation was also observed, further justifying the decision to proceed with Whipple surgery to prevent the potential malignancy of the mucinous adenoma.

## 5 Conclusion

XGP is a rare and challenging differential diagnosis for pancreatic neoplasms. Given its potential to mimic malignant lesions, a high index of suspicion is necessary. Echoendoscopy with fine-needle aspiration biopsy should be considered as a routine diagnostic tool before undertaking major surgery such as the Whipple procedure. Further studies are needed to better understand the etiology and optimal treatment of Xanthogranulomatous pancreatitis.

## Consent

Written informed consent was obtained from the patient for publication of this case report and accompanying images. A copy of the written consent is available for review by the Editor-in-Chief of this journal on request.

## Ethical approval

Given that this publication is a case report that does not contain identifiable patient information, this publication is exempt from ethical approval by the Institutional Ethics Committee.

## Funding

No source to be stated.

## Author contribution

Abdala Bolcatto, Melisa Erina, MD: data collection, writing the paper (First author).

Mandojana, Facundo Ignacio, MD: writing the paper (Corresponding author).

Verberck Simondi, Andres Vladimir, MD: data collection.

Viscido, German Rodrigo, MD: writing the paper.

## Guarantor

Abdala Bolcatto, Melisa Erina.

Viscido, German Rodrigo.

## Conflict of interest statement

The authors declare no conflicts of interest.

## References

[1] Gaur K., Mandal S., Mahajan N., Saluja S., Godhi S. (2019). Xanthogranulomatous pancreatitis-a rare case defying clinical, radiological and tumor marker diagnostics with a review of literature. Turk. Patol. Derg.

[2] Becker-Weidman D., Floré B., Mortelé K.J. (2017). Xanthogranulomatous pancreatitis: a review of the imaging characteristics of this rare and often misdiagnosed lesion of the pancreas. Clin. Imaging.

[3] Atreyapurapu V., Keshwani A., Lingadakai R., Pai K. (2016). Xanthogranulomatous pancreatitis mimicking a malignant solid tumour. Case Rep..

[4] Shima Y., Saisaka Y., Furukita Y., Nishimura T., Horimi T., Nakamura T. (2008). Resected xanthogranulomatous pancreatitis. J. Hepato-Biliary-Pancreat. Surg..

[5] Navarro Belén, Sáez-González Esteban, Ortuño Juan (2017). Xanthogranulomatous pancreatitis: a lesion that mimics pancreatic cancer. Rev. Esp. Enferm. Dig..

[6] Sohrabi C., Mathew G., Maria N., Kerwan A., Franchi T., Agha R.A. (2023). The SCARE 2023 guideline: updating consensus Surgical CAse REport (SCARE) guidelines. Int. J. Surg. Lond. Engl..

[7] Hanna T., Abdul-Rahman Z., Greenhalf W., Farooq A., Neoptolemos J.P. (2016). Xanthogranulomatous pancreatitis associated with a mucinous cystic neoplam. Pathol. Int..

[8] Betancourt Sosa Á.F., Padrón Sanabria J.A., Cedeño Miranda M.O., Barrios Evies A.J., González Larez C.A., Gregor Avendaño P.M. (2022). Cistoadenoma mucinoso de cuerpo y cola de páncreas: a propósito de un caso. Rev. Venez. Cir..

[9] Chao-González L., Barroso-Márquez L., Cepero-Valdés M., Moret-Vara S., Torres-Hernández Y.E., Torres-Rodríguez T.M. (2018). Valor del ultrasonido endoscópico para el diagnóstico diferencial de neoplasias pancreáticas. Revista Cubana de Cirugía..

[10] Recabarren M.G., Schlottmann F., Angeramo C.A., Kerman Cabo J., Casas M.A., Mella J. (2021). Ecoendoscopia en la estadificación del cáncer de esófago y de estómago. Rev. Argent. Cir..

[12] Kwon J.H., Kim J.H., Kim S.Y. (2018). Imaging and clinical features of xanthogranulomatous pancreatitis: an analysis of 10 cases at a single institution. Abdom. Radiol..

